# Supplementary material for: Trends in Airborne Chrysotile Asbestos Fibre Concentrations in Asbestos Cement Manufacturing Factories in Zimbabwe from 1996 to 2016
Source: Int J Environ Res Public Health. 2021 Oct 13;18(20):10755. doi: 10.3390/ijerph182010755 (PMC8535792; doi:10.3390/ijerph182010755)
Supplement: Supplementary file 1 [file ijerph-18-10755-s001.zip › ijerph-1345740-supplementary.pdf]

Supplementary Information

Trends in Occupational Exposure to Chrysotile Asbestos Fibre in Asbestos Cement Manufacturing Factories in Zimbabwe, 1996 to 2016,

Benjamin Mutetwa[1,*], Dingani Moyo[1,2] Derk Brouwer[1]
[1]School of Public Health, Faculty of Health Sciences, University of the Witwatersrand, Johannesburg, South Africa
[2] Faculty of Medicine and Health Sciences, Midland State University, Gweru, Zimbabwe
* Correspondence: bjmtetwa@yahoo.com; Tel.; +263 773 429 83

Content

1. One-Way ANOVA Output for various operational areas for the Harare and Bulawayo factory
2. Table S1: Number of chrysotile fibre personal sampling point measurements per operational area per year in the chrysotile cement manufacturing factories: 1996-2016.
3. Table S2: Number of monthly mean chrysotile fibre personal concentrations by factory, operational area and year in the chrysotile asbestos cement manufacturing factories: 1996 – 2016.
4. Tables S3 (a) to (c): Linear and multiple Regression modelling of personal exposure experience by operators at various operational areas by year and time period: Harare Factory
5. Tables S4 (a) – (d): Linear and multiple Regression modelling of personal exposure experience by operators at various operational areas by year and time period: Bulawayo Factory.
6. Tables S4 (a) and 5(b): Logistics regression modelling to examine whether year and time period have effect on personal exposure exceeding the OEL limit of 0.1 f/ml: Harare Factory.

NB: The number of personal samples decreased during the latter years, especially from 2008 to 2016, largely due to limited availability of consumables required for personal sampling of chrysotile asbestos fibre in various operational areas. Personal sampling at sheeting plant mixer, fettling table, kollergang and moulded goods was done at 2 or 3 operational points per month. For the pipe section, personal sampling data was collected at 2-4 joints lathe machines and at 2-3 full length pipe lathe machines per month. For multi-cutter operational tasks, personal sampling was done generally once per month where feasible.

Table S1: Number of chrysotile fibre personal sampling point measurements per operational area per year in the chrysotile cement manufacturing factories:

	1996	1997	1998	1999	2000	2001	2002	2003	2004	2005	2006	2007	2008	2009	2010	2011	2012	2013	2014	2015	2016	Total	
HARARE CHRYSOTILE CEMENT FACTORY	143	132	148	143	120	89	101	99	71	104	81	77	32	44	44	51	56	36	40	45	18	1674	
Saws cutting operations	63	69	72	71	60	42	40	35	25	35	26	23	12	16	17	20	23	18	19	20	7	713	
Fettling tables-scrapping and polishing	7	10	12	12	12	11	11	12	9	12	6	11	1	Nil	Nil	Nil	Nil	Nil	Nil	Nil	Nil	126	
Kollergang	41	19	17	20	14	14	19	17	11	21	16	14	6	8	9	15	12	10	9	7	2	301	
Moulded Goods	10	22	23	16	12	11	9	12	10	12	12	11	5	8	6	7	9	4	6	8	4	217	
Ground Hard Waste	11	12	12	12	10	Nil	11	11	5	12	9	8	Nil	5	7	6	12	4	6	10	5	168	
Laundry Room	11	Nil	12	12	12	11	11	12	11	12	12	10	8	7	5	3	Nil	Nil	Nil	Nil	Nil	149	
TOTAL	143	132	148	143	120	89	101	99	71	104	81	77	32	44	44	51	56	36	40	45	18	1674	
BULAWAYO CHRYSOTILE CEMENT FACTORY	54	114	179	187	117	88	50	119	108	Nil	53	80	16	14	25	22	1	12	6	4	25	1216	
(a) Sheeting Plant	37	75	100	105	65	52	28	70	56	Nil	27	43	9	14	20	10	1	8	6	3	25	754	
Saws cutting operations	21	38	54	59	39	33	16	42	33	Nil	17	23	6	6	5	Nil	Nil	3	3	2	16	416	
Fettling tables-scrapping and polishing	5	7	10	11	7	4	3	Nil	2	Nil	1	1	Nil	Nil	Nil	Nil	Nil	Nil	Nil	Nil	Nil	51	
Kollergang	Nil	8	11	9	8	6	3	19	20	Nil	8	13	3	5	10	8	1	4	3	Nil	5	144	
Ground Hard Waste	8	16	16	17	6	4	4	5	1	Nil	Nil	2	Nil	Nil	1	Nil	Nil	Nil	Nil	1	4	85	
(b) Pipe Plant (P/P)	17	39	79	82	52	36	22	49	52	Nil	26	37	7	Nil	5	12	Nil	4	Nil	1	Nil	520	
P/P-Lathe machining of pipe joints	10	17	37	47	32	21	12	22	20	Nil	11	17	3	Nil	2	6	Nil	2	Nil	1	Nil	260	
P/P-Lathe machining full length pipes	5	15	36	28	16	9	8	20	23	Nil	10	13	4	Nil	3	4	Nil	2	Nil	Nil	Nil	196	
Multicutter	2	7	6	7	4	6	2	7	9	Nil	5	7	Nil	Nil	Nil	2	Nil	Nil	Nil	Nil	Nil	64	
TOTAL																							2890

Table S2: Number of monthly mean chrysotile fibre personal concentrations by factory, operational area and year in the chrysotile asbestos cement manufacturing factories: 1996 - 2016

	1996	1997	1998	1999	2000	2001	2002	2003	2004	2005	2006	2007	2008	2009	2010	2011	2012	2013	2014	2015	2016	
FACTORY AND OPERATIONAL AREA																						
**HARARE CHRYSOTILE CEMENT FACTORY**	**60**	**56**	**72**	**72**	**70**	**59**	**65**	**71**	**54**	**71**	**62**	**60**	**28**	**37**	**35**	**38**	**42**	**29**	**32**	**35**	**15**	**1063**
Saws cutting operations	12	12	12	12	12	11	12	12	11	12	12	10	8	9	9	11	12	11	11	10	4	
Fettling tables-scrapping and polishing	7	10	12	12	12	11	11	12	9	12	6	11	1	Nil	Nil	Nil	Nil	Nil	Nil	Nil	Nil	
Moulded Goods	8	12	12	12	12	11	9	12	10	12	12	11	5	8	6	7	9	4	6	8	4	
Kollergang	11	10	12	12	12	11	11	12	8	11	11	10	6	8	8	11	9	10	9	7	2	
Ground Hard Waste	11	12	12	12	10	4	11	11	5	12	9	8	Nil	5	7	6	12	4	6	10	5	
Laundry Room	11	0	12	12	12	11	11	12	11	12	12	10	8	7	5	3	Nil	Nil	Nil	Nil	Nil	
**TOTAL**	**60**	**56**	**72**	**72**	**70**	**59**	**65**	**71**	**54**	**71**	**62**	**60**	**28**	**37**	**35**	**38**	**42**	**29**	**32**	**35**	**15**	**1063**
**BULAWAYO CHRYSOTILE CEMENT FACTORY**	**35**	**61**	**82**	**83**	**54**	**45**	**26**	**55**	**57**	**Nil**	**26**	**39**	**12**	**11**	**19**	**20**	**1**	**12**	**5**	**3**	**12**	**600**
**(a) Sheeting Plant**	**23**	**41**	**52**	**52**	**34**	**25**	**16**	**29**	**26**	**Nil**	**11**	**21**	**5**	**11**	**14**	**9**	**1**	**8**	**5**	**3**	**12**	**398**
Saws cutting operations	8	11	11	12	8	8	4	10	12	Nil	5	7	3	3	1	Nil	Nil	3	2	2	3	416
Fettling tables-scrapping and polishing	5	7	10	11	7	4	3	Nil	2	Nil	1	1	Nil	Nil	Nil	Nil	Nil	Nil	Nil	Nil	Nil	51
Kollergang	Nil	8	11	9	8	5	3	10	11	Nil	4	7	2	5	8	7	1	4	3	Nil	5	111
Ground Hard Waste	7	9	11	11	6	3	4	5	1	Nil	Nil	2	Nil	Nil	1	Nil	Nil	Nil	Nil	1	4	65
**(b) Pipe Plant (P/P)**	**12**	**20**	**30**	**31**	**20**	**20**	**10**	**26**	**31**	**Nil**	**15**	**18**	**7**	**Nil**	**5**	**11**	**Nil**	**4**	**Nil**	**Nil**	**Nil**	**260**
P/P-Lathe machining of pipe joints	6	6	12	12	8	7	4	9	11	Nil	5	7	3	Nil	2	5	Nil	2	Nil	Nil	Nil	99
P/P-Lathe machining full length pipes	4	7	12	12	8	7	4	10	11	Nil	5	4	4	Nil	3	4	Nil	2	Nil	Nil	Nil	97
Multicutter	2	7	6	7	4	6	2	7	9	Nil	5	7	Nil	Nil	Nil	2	Nil	Nil	Nil	Nil	Nil	64
**TOTAL**																						**1663**

One-Way ANOVA Output for various operational areas for the Harare and Bulawayo factory

(a) Harare factory

Saws operator ($F(2, 248) = 880$, $p < 0.001$); moulded goods operator ($F(2, 248) = 127$, $p< 0.001$); kollergang ($F(2, 248) = 173$, $p < 0.001$); ground hard waste operator ($F(2, 248) = 231$, $p < 0.001$); laundry room operator ($F(2, 248) = 243$, $p < 0.001$; overall factory mean ($F(2, 248) = 484$, $p < 0.001$), $F(2, 248) = 0.25$, $p = 0.78$).

(b) Bulawayo factory

Saws cutting operator $F(2, 238) = 579.9$, $p<0.001$); fettling table operator $F(2, 238) = 463.6$, $p <0.001$); kollergang operator $F(2, 238) = 298.3$, $p<0.001$); ground hard waste operator $F(2, 238) = 171.6$, $p<0.001$; pipe joints operator $F(2, 238) = 220.0$, $p<0.001$); full length pipe operator $F(2, 238) = 205.5$, $p<0.001$); multi-cutter operator $F(2, 238) = 48.4$, $p<0.001$); overall factory $F(2, 238) = 375.3$ $p<0.001$).

Table S3. a: Linear and multiple Regression modelling of personal exposure experience by operators at various operational areas by year and time-period: Harare Factory.

**Model variable**	**Model 1[a]**				**Model 2[b]**			
	β	$StdE^{\beta}$	p-value	**Confidence Interval** LB UB	β	$StdE^{\beta}$	p-value	**Confidence Interval** LB UB
**Saw cutting operations**								
▪ Year	-0.008	0.000	<0.001	-0.008 - 0.007	-0.004	0.000	<0.001	-0.005 0.003
▪ Time period	-0.06	0.001	<0.001	-0.063 - 0.057	-0.031	0.004	<0.001	-0.038 -0.024
▪ R							0.950	
▪ R^2							0.902	
▪ R^2 adjusted							0.901	
▪ StdE							0.016	
▪ F (p-value)							1135.694 (<0.001)	
**Fettling table operations**								
▪ Year	0.000	0.000	0.475	-0.001 0.000	0.000	0.001	0.825	-0.001 -0.001
▪ Time period	-0.001	0.002	0.496	-0.005 0.002	0.000	0.005	0.972	-0.010 -0.010
▪ R							0.045	
▪ R^2							0.002	
▪ R^2 adjusted							-0.006	
▪ StdE							0.023	
▪ F (p-value)							0.255 (0.775)	

[a] Simple Linear Regression model for each variable and chrysotile fibre personal exposure, [b] Multiple linear regression for all variables, StdE – standard error of β coefficient,
LB– lower bound, UB – upper bound.

Table S3. b: Linear and multiple Regression modelling of personal exposure experience by operators at various operational areas by year and time-period: Harare Factory cont'd.

Model variable	Model 1[a]				Model 2[b]			
	β	StdE$^{\beta}$	p-value	Confidence Interval LB UB	β	StdE$^{\beta}$	p-value	Confidence Interval LB UB
**Moulded goods operations**								
▪ Year	-0.004	0.000	<0.001	-0.005 -0.003	-0.004	0.001	<0.001	-0.006 -0.003
▪ Time period	-0.029	0.002	<0.001	-0.034 -0.024	0.000	0.006	0.984	-0.012 0.013
▪ R							0.660	
▪ R^2							0.436	
▪ R^2 adjusted							0.431	
▪ StdE							0.028	
▪ F (p-value)							95.785 (<0.001)	
**Kollergang operations**								
▪ Year	-0.005	0.000	<0,001	-0.005 -0.004	-0.005	0.001	<0.001	-0.006 -0.003
▪ Time period	-0.034	0.007	<0.001	-0.038 -0.030	0.001	0.005	0.813	-0.009 0.011
▪ R							0.779	
▪ R^2							0.607	
▪ R^2 adjusted							0.604	
▪ StdE							0.023	
▪ F (p-value)							191.687 (<0.001)	

[a] Simple Linear Regression model for each variable and chrysotile fibre personal exposure, [b] Multiple linear regression for all variables, StdE – standard error of
β coefficient, LB – lower bound, UB – upper bound.

Table S(3c): Linear and multiple Regression modelling of personal exposure experience by operators at various operational areas by year and time-period: Harare Factory cont'd

Model variable	Model 1[a]				Model 2[b]			
	β	StdE$^{\beta}$	p-value	Confidence Interval	β	StdE$^{\beta}$	p-value	Confidence Interval

				**UB LB**				**UB LB**
**Ground hard waste operations**								
▪ Year	-0.006	0.000	<0.001	-0.006 -0.005	-0.006	0.001	<0.001	-0.007 -0.004
▪ Time period	-0.042	0.002	<0.001	-0.046 -0.038	-0.002	0.005	0.710	-0.012 0.008
▪ R							0.844	
▪ R^2							0.712	
▪ R^2 adjusted							0.710	
▪ StdE							0.022	
▪ F (p-value)							307.141(<0.001)	
**Laundry operations**								
▪ Year	-0.005	0.000	<0.001	-0.006 -0.005	-0.004	0.001	<0.001	-0.006 -0.003
▪ Time period	-0.038	0.002	<0.001	-0.042 -0.034	-0.005	0.005	0.031	-0.016 0.005
▪ R							0.807	
▪ R^2							0.651	
▪ R^2 adjusted							0.648	
▪ StdE							0.023	
▪ F (p-value)							230.951(<0.001)	
**Overall Factory**								
▪ Year	-0.069	0.003	<0.001	-0.025 -0.064	-0.005	0.000	<0.001	-0.005 -0.0004
▪ Time period	-0.526	0.023	<0.001	-0.571 -0.480	-0.007	0.004	0.047	-0.014 0.000
▪ R							0.906	
▪ R^2							0.820	
▪ R^2 adjusted							0.819	
▪ StdE							0.015	
▪ F (p-value)							565.112(<0.001)	

[a] Simple Linear Regression model for each variable and chrysotile fibre personal exposure, [b] Multiple linear regression for all variables, StdE – standard error of β coefficient, LB – lower bound, UB – upper bound, R – correlation coefficient.

Table S4(a): Linear and multiple Regression modelling of personal exposure experience by operators at various operational areas by year and time-period: Bulawayo factory

Model variable	Model 1[a]				Model 2[b]			
	β	$StdE^{\beta}$	p-value	Confidence Interval LB UB	β	$StdE^{\beta}$	p-value	Confidence Interval LB UB
**Saw cutting operations**								
▪ Year	-0.007	0.000	<0.001	-0.008 -0.007	-0.006	0.000	<0.001	-0.007 -0.005
▪ Time period	-0.055	0.002	<0.001	-0.058 -0.052	-0.009	0.003	<0.007	-0.015 -0.003
▪ R							0.955	
▪ R^2							0.912	
▪ R^2 adjusted							0.912	
▪ StdE							0.014	
▪ F (p-value)							1235.4 (<0.001)	
**Fettling table operations**								
▪ Year	-0.013	0.000	<0.001	-0.014 -0.013	-0.014	0.001	<0.001	-0.015 -0.012
▪ Time period	-0.095	0.003	<0.001	-0.101 0.089	-0.006	0.006	0.281	-0.005 0.017
▪ R							0.958	
▪ R^2							0.918	
▪ R^2 adjusted							0.918	
▪ StdE							0.024	
▪ F (p-value)							1331.5 (<0.001)	

[a] Simple Linear Regression model for each variable and chrysotile fibre personal exposure, [b] Multiple linear regression for all variables, StdE – standard error of β coefficient, LB – lower bound, UB – upper bound.

Table S4(b): Linear and multiple Regression modelling of personal exposure experience by operators at various operational areas by year and time-period: Bulawayo Factory cont'd.

Model variable	Model 1[a]				Model 2[b]			
Operator	β	$StdE^{\beta}$	p-value	**Confidence Interval**	β	$StdE^{\beta}$	p-value	**Confidence Interval**

				LB UB				LB UB
**Kollergang**								
▪ Year	-0.005	0.000	<0.001	-0.006 -0.005	-0.005	0.000	<0.001	-0.006 -0.004
▪ Time period	-0.06	0.002	<0.001	-0.041 -0.035	0.001	0.004	0.840	-0.006 -0.008
▪ R							0.889	
▪ R^2							0.807	
▪ R^2 adjusted							0.806	
▪ StdE							0.016	
▪ F (p-value)							496.62 (<0.001)	
**Ground hard waste**								
▪ Year	0.004	0.000	<0.001	-0.005 -0.004	-0.005	0.001	<0.001	-0.006 -0.004
▪ Time period	-0.030	0.002	<0.001	-0.033 -0.027	0.004	0.004	0.295	-0.004 -0.012
▪ R							0.834	
▪ R^2							0.696	
▪ R^2 adjusted							0.693	
▪ StdE							0.017	
▪ F (p-value)							270.79 (<0.001)	

[a] Simple Linear Regression model for each variable and chrysotile fibre personal exposure, [b] Multiple linear regression for all variables, StdE – standard error of β coefficient, LB – lower bound, UB – upper bound.

Table S4(c): Linear and multiple Regression modelling of personal exposure experience by operators at various operational areas by year and time-period: Bulawayo Factory cont'd.

Model variable			Model 1[a]				Model 2[b]	
**Operator**	β	StdE[β]	p-value	**Confidence Interval**	β	StdE[β]	p-value	**Confidence Interval**
				LB UB				LB UB
**Pipe joints**								
▪ Year	-0.005	0.000	<0.001	-0.005 -0.004	0.001	0.001	<0.001	-0.006 -0.004
▪ Time period	-0.034	0.002	<0.001	-0.038 -0.031	0.004	0.004	0.773	-0.007 0.009
▪ R							0.855	
▪ R^2							0.731	
▪ R^2 adjusted							0.728	
▪ StdE							0.018	

▪ F (p-value)							321.47 (<0.001)	
**Full length pipe**								
▪ Year	-0.004	0.000	<0.001	-0.005 -0.004	-0.004	0.001	<0.001	-0.005 -0.003
▪ Time period	-0.032	0.002	<0.001	-0.035 -0.028	-0.002	0.004	0.671	-0.010 0.006
▪ R							0.838	
▪ R^2							0.701	
▪ R^2 adjusted							0.699	
▪ StdE							0.017	
▪ F (p-value)							278.44 (<0.001)	

[a] Simple Linear Regression model for each variable and chrysotile fibre personal exposure, [b] Multiple linear regression for all variables, StdE – standard error of β coefficient, LB – lower bound, UB – upper bound.

Table S4(d): Linear and multiple Regression modelling of personal exposure experience by operators at various operational areas by year and time-period: Bulawayo cont'd.

**Model variable**			**Model 1[a]**				**Model 2[b]**	
**Operator**	β	StdE$^{\beta}$	p-value	**Confidence Interval** LB UB	β	StdE$^{\beta}$	p-value	**Confidence Interval** LB UB
**Multi-cutter**								
▪ Year	-0.001	0.000	<0.001	-0.002 -0.001	-0.001	0.000	0.002	-0.002 0.000
▪ Time period	-0.011	0.001	<0.001	-0.013 -0.008	-0.002	0.003	0.620	-0.008 0.005
▪ R							0.553	
▪ R^2							0.306	
▪ R^2 adjusted							0.300	
▪ StdE							0.013	
▪ F (p-value)							52.29 (<0.001)	
**Overall factory**								
▪ Year	-0.005	0.000	<0.001	-0.006 -0.005	-0.005	0.000	<0.001	-0.006 -0.004
▪ Time period	-0.039	0.001	<0.001	-0.042 - 0.039	-0.003	0.003	0.339	-0.010 0.003
▪ R							0.915	
▪ R^2							0.837	
▪ R^2 adjusted							0.835	
▪ StdE							0.014	

▪ F (p-value)	607.80 (<0.001)

[a] Simple Linear Regression model for each variable and chrysotile fibre personal exposure, [b] Multiple linear regression for all variables, StdE – standard error of β coefficient, LB – lower bound, UB – upper bound.

Table S5(a): Logistics regression modelling to examine whether year and time-period have effect on personal exposure exceeding the OEL limit of 0.1 f/ml: **Harare Factory.**

Parameter	NagR²	% OPAC	β	Wald- χ²(p-value)	OR (95% CI)
Saw cutting operations	0.860	93.6			
▪ Year			-0.597	11.958 (0.001)	0.551 (0.393 - 0.772)
▪ Time period			-1.887	3.826 (0.05)	0.152 (0.023 - 1.004)
Fettling table operations	0.284	88.0			
▪ Year			0.067	0.325 (0.569)	1.070 (0.849 – 1.348)
▪ Time period			1.408	2.650 (0.103)	4.089 (0.752 – 22.228)
Moulded goods	0.412	75.7			
operations			-0.389	22.043 (<0.001)	0.678
▪ Year			-1.110	3.791 (0.052)	(0.576-0.797)
▪ Time period					3.035 (0.993 – 9.280)
Kollergang operations	0.600	84.9			
▪ Year			-0.447	23.043 (<0.001)	0.639 (0.533 - 0.768)
▪ Time period			0.628	0.971 (0.324)	3.035 (0.993 – 9.280
Ground hard waste	0.793	95.2			
▪ Year			-0.243	4.524 (0.033)	0.785 (0.627-0.981)
▪ Time period			-2.923	11.122 (0.001)	0.537 (0.537-6.536)
Laundry Room	0.651	88.0			
▪ Year			-0.382	16.725 (<0.001)	0.683 (0.569-0.583)
▪ Time Period			-0.293	0.192 (0.662)	0.293 (0.021-4.067)
Overall Harare factory	0.911	97.2			
▪ Year			-1.138	13.906 (<0.001)	0.321(0.176-0.820)
▪ Time Period			-1.227	0.836 (0.662)	0.746 (0.201-2.769)

NagR² - Nagelkerke R Square, OPAC- Overall percentage accuracy in classification, β- coefficient, OR – Odds Ratio, CI-Confidence limit.

Table S5(b): Logistics regression to examine year and time-period's effect on personal exposure exceeding the OEL limit of 0.1 f/ml: Factory. Bulawayo factory

Parameter	NagR²	% OPAC	β	Wald- χ² (p-value)	OR (95% CI)
**Saw cutting**	0.876	92.5			
**operations**			-0.583	17.17 (<0.001)	0.558 (0.424 -
▪ Year			-17.99	0.000	0.735)
▪ Time period				(<0.001)	0.000 (0.000 - -)
**Fettling table**	0.891	96.3			
**operations**			1.830	21.156 (<0.001)	0.160 (0.074 –
▪ Year			5.406	11.38 (0.001)	0.350)
▪ Time period					

					222.845 (9.633 –
					51555.11)
**Kollergang**	0.891	96.3			
**operations**			1.830	21.156 (<0.001)	0.160 (0.074 –
▪ Year			5.406	11.38 (0.001)	0.350)
▪ Time period					222.845 (9.633 –
					51555.11)
**Ground Hard Waste**	0.720	84.6			
**operations**			-0.958	37.629 (<0.001)	0.384 (0.283 -
▪ Year			3.475	16.045 (<0.001)	0.521)
▪ Time period					32.291 (5.898 –
					176.797)
**Pipe Joints**	0.150	98.3			
**operations**			0.031	0.011 (0.916)	1.032 (0.573-
▪ Year			-2.052	1.052 (0.372)	1.858)
▪ Time Period					0.128 (0.001-
					11.628)
**Full length pipe**	0.723	87.9			
**operations**			-0.513	23.264 (<0.001)	0.599 (0.486-
▪ Year			0.284	0.149 (0.699)	0.738)
▪ Time Period					1.328 (0.314-
					5.617)
**Multi-cutter**	0.091	95.0			
**operations**			0.133	0.743 (0.389)	1.143 (0.844-
▪ Year			0.066	0.003	1.547)
▪ Time Period				(0.953)	1.068 (0.118-
					9.676)
**Overall Bulawayo**	0.827	95.4			
**factory**			-0.616	21.410(<0.001)	0.540 (0.416-
▪ Year			-0.599	0.442 (0.506)	0.701)
▪ Time Period					0.549 (0.094-
					3.215)

NagR2-Nagelkerke R-Square, OPAC-Overall percentage accuracy in classification, β-coefficient, OR–Odds Ratio, CI-Confidence limit.
